# Personal

**Published:** 1898-11

**Authors:** 


### PERSONAL.

DR. WILLIAM C. KRAUSS, of Buffalo, who suffered from a severe illness during a portion of the summer, has recovered and resumed the practice of his profession. He presided at the recent meeting of the Medical Association of Central New York, at Auburn, and his address, which merits reading, is publised in the current issue of the JOURNAL.

Drs. Edward Clark and William B. May, of Buffalo, on recommendation of Health Commissioner Wende, were recently appointed special patrolmen by the Police Commissioners, for the purpose of enforcing the milk ordinances of the city. These are excellent appointments and this plan indicates further zealous care of the milk supply, which has always received the special attention of Dr. Wende.

---

Dr. Frederick Peterson, of New York, has removed from 60 W. 50th to 4 W. 50th Street. Office hours, 9-12 o'clock. Telephone, 779, 38th Street.

